# The Association between Peer Victimization and Risk-Taking Behavior among Chinese Adolescents: Testing a Moderated Mediation Model

**DOI:** 10.3390/ijerph192114198

**Published:** 2022-10-30

**Authors:** Yangang Nie, Guodong Wang, Pei Chen, Linxin Wang, Kai Dou

**Affiliations:** 1 Department of Psychology and Research Center of Adolescent Psychology and Behavior, School of Education, Guangzhou University, Guangzhou 510006, China; 2 Beijing Key Laboratory of Applied Experimental Psychology, National Demonstration Center for Experimental Psychology Education (Beijing Normal University), Institute of Developmental Psychology, Beijing Normal University, Beijing 100875, China

**Keywords:** peer victimization, risk-taking behavior, self-control, positive parenting, adolescent

## Abstract

Peer victimization has been considered a main source of risk-taking behavior among adolescents, but little is known about the mechanisms underlying this association. Based on the social–cognitive theory and the person–environment interactions model, the current study built a moderated mediation model to explore whether self-control mediated the link between peer victimization and adolescent risk-taking behavior and whether positive parenting moderated this link. We used a 2-time longitudinal design (6 months apart) to investigate 488 adolescents (*M*_age_ = 15.63 years, *SD* = 1.64) from 3 middle schools in Guangzhou. The results were as follows: (1) There were significant correlations among peer victimization, adolescent risk-taking behavior, self-control, and positive parenting when controlling for demographic variables. (2) Peer victimization not only influenced risk-taking behavior directly, but also indirectly through self-control. (3) Positive parenting moderated the influence of self-control on risk-taking behavior. In other words, positive parenting could enhance the inhibitory effect of self-control on risk-taking behavior. The results help reveal the mechanism by which adolescent risk-taking behavior forms and may help inform interventions against adolescent risk-taking behavior.

## 1. Introduction

Risk-taking behavior refers to individuals participating in behaviors with potential risks or negative consequences in pursuit of positive results or benefits [1]. Adolescence is a period of heightened potential to engage in risk-taking behavior [2], such as taking dangerous drugs, conducting minor criminal activity, and participating in alcohol abuse. Indeed, risk-taking behavior as a public health challenge has been linked to severe negative outcomes [3,4]. Given the detrimental effects of risk-taking on developmental outcomes, understanding the mechanisms by which adolescents are likely to engage in risk-taking behavior is essential.

Recent studies have confirmed that peer victimization contributes to risk-taking behavior [5,6,7]. Peer victimization is conceptualized as the experience of being bullied by or receiving aggression from peers [8]. Compared to children, adolescents show heightened levels of sensitivity and responsiveness to peer feedback and rejection [9,10]. According to the social bonding theory [11], peer victimization weakens adolescents’ social bonding, which is not conductive to the development of social norms and, thus, increases risk-taking behaviors (e.g., substance use, aggression, and gambling). Consistent with this theory, Jiang et al. found that adolescents who suffered more peer victimization exhibited more substance use [5]. Despite these findings, the mediating and moderating variables involved in this relationship remain unclear. Exploring these factors is necessary to inform more effective interventions aimed at reducing adolescent risk-taking behavior. To address these gaps, the first aim of this study was to examine whether self-control serves as a mediator in the link between peer victimization and adolescent risk-taking behavior. The second aim of this study was to examine whether positive parenting acts as a protective factor that moderates this mediating process.

### 1.1. Mediating Role of Self-Control

Self-control is the ability to withstand impulses, conform oneself to social norms, and support the pursuit of long-term goals [12,13]. In alignment with the social–cognitive theory [14], the environment affects people’s behavior through their cognitions, and self-control seems to be a plausible cognitive mediator of the link between peer victimization and adolescents’ risk-taking behavior. Specifically, peer victimization, as an important aspect of an adolescent’s environment, may decrease self-control and increase adolescent risk-taking behavior.

From one perspective, adolescents who experienced peer victimization may be predisposed to low levels of self-control [15]. As a source of pressure, peer victimization consumes adolescents’ cognitive resources, weakening their self-control [16,17]. Equal communication and interaction between adolescents and their peers can help them form a sense of social norms. In this process, adolescents restrain and imitate each other, which promotes a high level of self-control [18]. Previous studies have verified a negative association between peer victimization and self-control [6,15,19]. For example, Telzer et al. demonstrated that victimized adolescents showed greater activation in regions involved in self-control [6]. Similarly, in a sample of 1849 Chinese adolescents, Wang et al. found that peer victimization negatively predicted lower levels of self-control [19]. Furthermore, self-control may further contribute to adolescent risk-taking behavior. Adolescents with low levels of self-control may show a poor ability to inhibit impulses and delay gratification, resulting in more risk-taking behaviors [3]. Meanwhile, adolescents with high levels of self-control are inclined to consider the long-term consequences of their current behaviors; thus, they are less likely to be involved in risk-taking behavior [20,21]. Prior studies have consistently found that self-control is robustly associated with risk-taking behavior [3,22,23].

Although there is indirect evidence for the two separate paths from peer victimization to self-control and from self-control to adolescent risk-taking behavior, the possible mediating role of self-control between peer victimization and adolescent risk-taking behavior has yet to be tested directly. Directly testing the mediating role of self-control would enhance the extant knowledge base by illuminating a specific psychological mechanism through which peer victimization translates into the specific behavioral outcomes of adolescent risk-taking behavior. Therefore, we proposed the Hypothesis 1: self-control mediates the relationship between peer victimization and adolescent risk-taking behavior.

### 1.2. Moderating Role of Positive Parenting

Although exposure to peer victimization may increase risk-taking behavior via self-control, victimized adolescents may not all engage equally in risk-taking behavior. To understand the heterogeneity of adolescent risk-taking behavior, the person–environment interaction model identifies other environmental factors that interact with individual factors (e.g., self-control) to affect risk-taking behavior [24]. In other words, another environmental experience may moderate the relationship between self-control and risk-taking behavior. In this study, we examined modes of positive parenting.

Positive parenting refers to parental behaviors that are warm, supportive, and responsible, including, but not limited to, teaching, listening, expressing approval, and providing emotional support [25]. Numerous studies have revealed that positive parenting plays a pivotal role in individual adaptation, such as in problematic mobile phone use [26], smoking and drinking [27], and sexual risk-taking [28]. Furthermore, previous studies have found that parenting styles moderate the effect of self-control on adolescent development [26,29]. For instance, a study among Chinese adolescents found that the interaction between self-control and parental phubbing affected addictive behavior [26]. Positive parenting, such as parental behavioral control and parental knowledge, may provide fewer opportunities for adolescents with low self-control to engage in risk-taking behavior [10]. Based on the person–environment interaction model and previous research, we proposed the Hypothesis 2: positive parenting may boost the impact of self-control on risk-taking behavior.

### 1.3. The Present Study

**Figure 1:**
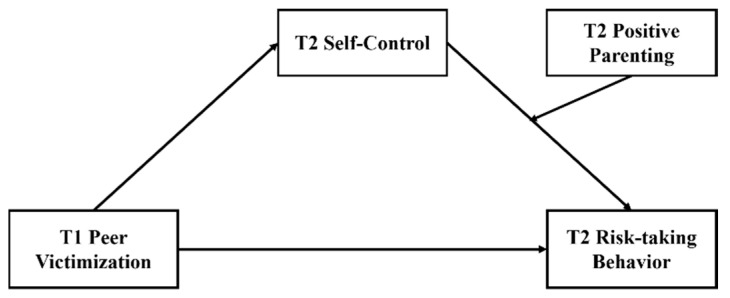
Conceptual Model. Note: T1 = Time 1, T2 = Time 2.

In summary, the purpose of this study was to investigate the mechanism underlying the influence of peer victimization on adolescent risk-taking behavior. We proposed a moderated mediation model (see Figure 1) to determine (a) whether self-control mediates the association between peer victimization and adolescent risk-taking behavior and (b) whether positive parenting moderates the path of self-control to risk-taking behavior.

## 2. Materials and Methods

### 2.1. Participants

Using a 2-time longitudinal design (6 months apart), this study recruited 488 adolescents (49.60% females, 51.40% males) and their parents (61.60% females, 38.40% males) by convenience sampling at T1 (June 2019) in Guangzhou, China. The average age of adolescents was 14.44 (*SD* = 1.55) and the average age of their parents was 41.61 (*SD* = 4.34). At T2 (December 2019), 448 adolescents (50.67% females; *M*_age_ = 14.46 years, *SD* = 1.56) and their parents (62.20% females; *M*_age_ = 41.65 years, *SD* = 4.34) remained in the study (attrition rate = 8.20%). Regarding parents’ level of education, 72.95% of the fathers and 68.03% of the mothers had graduated middle-school, 17.83% of the fathers and 12.30% of the mothers had a college degree or equivalent, and 0.82% of the fathers and 0.41% of the mothers had a postgraduate degree.

This research used a collective test method, taking class as a unit to investigate students who responded to self-report questionnaires. Before the research began, adolescent participants and their guardians and school administrators provided informed assent and informed consent, respectively. All collection procedures were approved by the Research Ethics Committee of Guangzhou University (protocol number: GZHU 2019012).

### 2.2. Measures

#### 2.2.1. Peer Victimization at T1

The four-item Peer-victimization Scale [30] was used to assess adolescents’ perceived peer victimization (e.g., “Have you ever been picked on or said mean things by your peers in the last six months”). Items were rated on a five-point scale (from “0 = never” to “4 = every day”). A higher mean score indicated a high level of perceived peer victimization. This scale has been demonstrated to be valid and reliable among Chinese adolescents [31]. McDonald’s omega was 0.64.

#### 2.2.2. Self-Control at T2

The 13-item Chinese version of the Brief Self-Control Scale (BSCS) was used to assess adolescents’ self-control (e.g., “I am good at resisting temptation”) [32]. Adolescents responded to the items using a five-point scale (from “1 = not like me at all” to “5 = like me very much”). A higher mean score indicated a higher level of self-control. This scale has been demonstrated to be valid and reliable among Chinese adolescents [3]. McDonald’s omega was 0.83.

#### 2.2.3. Positive Parenting at T2

The three-item revised Chinese version of the Positive Parenting Subscale from the Alabama Parenting Questionnaire [33] was used to assess positive parenting that adolescents are perceived to be receiving from their parents (e.g., “Your parents praise you for behaving well”). Items were rated on a five-point scale (from “1 = never” to “5 = always”). This scale has been demonstrated to be valid and reliable among Chinese adolescents [34]. McDonald’s omega was 0.94.

#### 2.2.4. Risk-Taking Behavior at T2

The 12-item Chinese version of the Adolescent Risk-Taking Questionnaire [35] was used to measure parents’ perceived risk-taking behavior (e.g., “How frequently do your kids smoke”) with their adolescent. Items were rated on a five-point scale (from “0 = never” to “4 = always”). This scale has been demonstrated to be valid and reliable among Chinese adolescents [3]. McDonald’s omega was 0.68.

#### 2.2.5. Control Variables

Considering its impact on risk-taking behavior, we collected participants’ demographic information at T1, including student gender (1 = male, 2 = female), student age, and mothers’ and fathers’ education (1 = primary school, 2 = middle school, 3 = undergraduate, 4 = postgraduate student) [3].

### 2.3. Statistical Analysis

First, we computed descriptive statistics and correlations among all variables using IBM SPSS 26.0. Second, we tested a mediation model using Mplus 8.3 [36] to examine Hypotheses 1 and 2. Using a bootstrapping (*N* = 5000) technique [37], we tested the mediating effects of T2 self-control on T1 peer victimization and T2 risk-taking behavior after controlling the impact of covariates on self-control and risk-taking behavior, respectively. Third, we integrated the moderator (i.e., positive parenting) into the mediation model to test Hypothesis 2. Across the path models, we addressed the missing data using full information maximum likelihood estimation [38].

## 3. Results

### 3.1. Descriptive Statistics

The descriptive statistics and bivariate correlations of the key variables and covariates are presented in Table 1. Specifically, T1 peer victimization was negatively associated with T2 self-control but positively associated with T2 risk-taking behavior. Moreover, T2 self-control was negatively associated with T2 risk-taking behavior but positively associated with T2 positive parenting.

### 3.2. Mediating Effects of Self-Control

**Figure 2:**
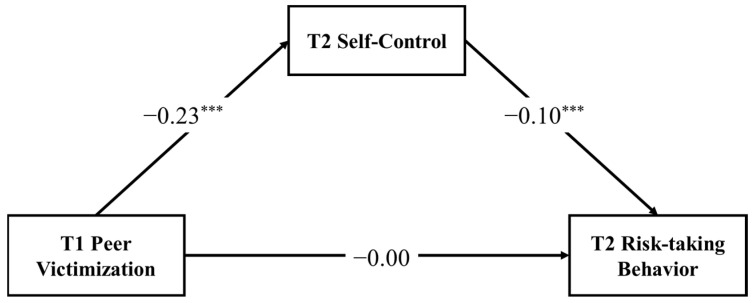
The mediating effect of self-control in the relationship between peer victimization and risk-taking behavior. Note: Unstandardized coefficients are reported. T1 = Time 1, T2 = Time 2; *** *p* < 0.001.

The mediation model depicted in Figure 2 exhibited saturated fit to the data. After controlling for covariates, T1 peer victimization was significantly associated with T2 self-control (*B* = −0.23, *SE* = 0.05, 95%CI = (−0.330, −0.121)). Our mediation analysis results (see Table 2) indicated that the mediation effect of T2 self-control (indirect effect = 0.02, *SE* = 0.01, 95% CI = (0.011, 0.039)) was significant.

### 3.3. Moderating Effects of Positive Parenting

**Figure 3:**
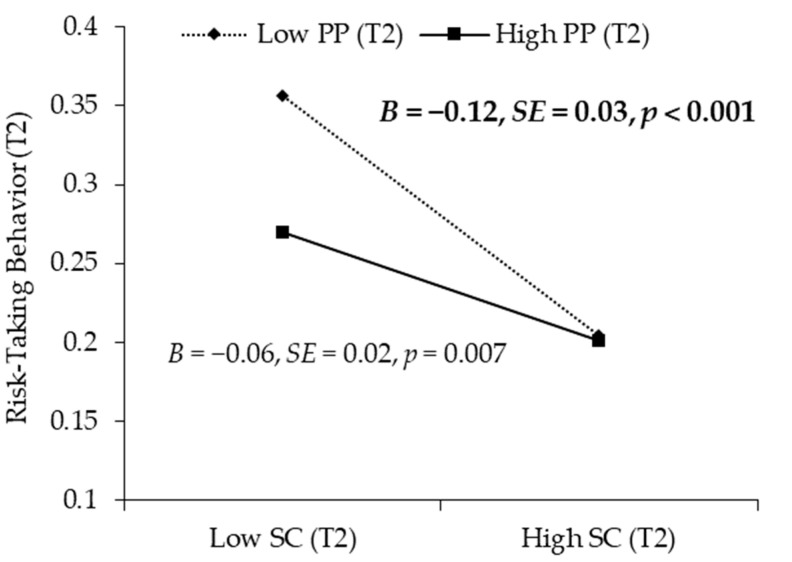
The relation between T2 self-control and T2 risk-taking behavior moderated by T2 positive parenting. Note: Unstandardized coefficients are reported. T1 = Time 1, T2 = Time 2; SC = self-control, PP = positive parenting.

Based on the mediation model, we continued to test whether T2 positive parenting would moderate the association between T2 self-control and T2 risk-taking behavior and to examine the extent to which positive parenting would moderate the mediation effect of self-control. The moderated mediation model fit the data well: χ^2^ = 11.43, *df* = 7, *p* = 0.121; RMSEA = 0.038, 90% CI = (0.000, 0.075); CFI = 0.960; SRMR = 0.023. As shown in Table 3, the results suggested that T2 positive parenting moderated the relationship between T2 self-control and T2 risk-taking behavior (*B* = 0.04, *SE* = 0.02, 95% CI = (0.000, 0.075)). A follow-up simple slopes test (Figure 3) indicated that the relationship between T2 self-control and T2 risk-taking behavior was stronger when T2 positive parenting was low (*B* = −0.12, *SE* = 0.03, 95% CI = (−0.195, −0.064)) than when T2 positive parenting was high (*B* = −0.06, *SE* = 0.02, 95% CI = (−0.097, −0.017)).

As shown in Table 4, the moderated mediation model suggested that the mediating effect of self-control was significantly stronger when T2 positive parenting was lower (*B* = 0.03, *SE* = 0.01, 95% CI = (0.012, 0.053)) than when T2 positive parenting was higher (*B* = 0.01, *SE* = 0.01, 95% CI = (0.003, 0.028)). In summary, peer victimization had a stronger positive relationship with adolescents’ risk-taking behavior via self-control when positive parenting was lower.

## 4. Discussion

Based on the social–cognitive theory and person–environment interactions model, this study prospectively examined the mediating (i.e., how peer victimization is associated with risk-taking behavior) and moderating mechanisms (i.e., when the harm is most potent) underlying the significant association between peer victimization and adolescent risk-taking behavior. The results indicated that self-control mediated the relationship between peer victimization and adolescent risk-taking behavior and that self-control also interacted with positive parenting to influence risk-taking behavior.

Our findings confirmed Hypothesis 1, that the positive relationship between peer victimization and adolescent risk-taking behavior is mediated by self-control. Many cross-sectional studies have identified self-control as a critical mediator linking negative environmental factors and risk-taking behavior [3,19,39]. The present study is the first to reveal a longitudinal association between peer victimization and adolescent risk-taking behavior. Consistent with the social–cognitive theory, self-control seems to be a plausible cognitive mediator of the link between key aspects of the peer victimization and adolescent risk-taking behavior [14]. For many adolescents, it is important to obtain acceptance from peers. In a normal environment, maintaining relationships with peers can help adolescents form behavioral principles consistent with social norms and develop a high level of self-control [3,18]. Conversely, adolescents who experienced peer victimization may be more likely to experience a range of aversive emotional states [40]. This will result in insufficient cognitive resources for adolescents to maintain their motivation to exert self-control and thus increase their risk-taking behaviors [3,16]. This finding highlighted that adolescents’ self-control can reduce risk-taking behavior caused by peer victimization.

In addition, our results supported Hypothesis 2. Positive parenting moderated the impact of self-control on risk-taking behavior. The magnitude of the path coefficient from self-control to risk-taking behavior was large for those adolescents with high levels of positive parenting. This result supports the person–environment interactions model, in which adolescent risk-taking behavior is affected by a complex interaction between individual factors and environmental experience [24]. Adolescents who experience positive parenting may receive more emotional warmth and social support; thus, they may be less likely to engage in risk-taking behaviors that are generally not allowed by their parents [41]. Moreover, even if adolescents have lower self-control, positive parenting, denoting higher parental behavioral control and knowledge, may reduce the chance of adolescents engaging in risk-taking behaviors [10]. Thus, positive parenting can moderate the impact of self-control on risk-taking behavior.

## 5. Implications and Limitations

The results of this study have several theoretical implications and practical implications. Using longitudinal investigations, the current research explained how peer victimization was associated with adolescent risk-taking behavior from the perspective of social–cognitive theory and the person–environment interaction model. The results expand the research themes of risk-taking behavior and help to further deepen the understanding of its mechanisms. In terms of practical implications, future prevention and intervention for peer victimization and risk-taking behaviors should be based on enhancing adolescent self-control and positive parenting behaviors. From one perspective, educators could enhance adolescent self-control through interventions to strengthen self-control traits or executive function, such as mindfulness training, approach-avoidance training, and exercise training [42,43]. From another perspective, parents should increase the use of positive parenting and strengthen their concern for their children’s daily lives, which, in turn, reduces adolescent risk-taking behaviors. In addition, parents and schoolteachers need to form a close home–school partnership to provide timely psychological guidance to adolescents who suffer from peer aggression to prevent risk-taking behaviors.

Although the present study advances our understanding of the relationship between peer victimization and adolescent risk-taking behavior, some limitations need to be considered. Firstly, there is the small sample size selected by the convenient sampling method in this study, which led to the low ecological validity of the results, that is, the poor generalization. Future studies need to enhance the representativeness of the sample to improve the ecological validity of the results. Second, the McDonald’s omega of the Adolescent Risk-Taking Questionnaire was relatively low, which may not adequately measure the adolescent risk-taking behavior. Therefore, future research may benefit from the use of more flexible instruments and multiple informants to collect data to provide a much stronger test of the model. Thirdly, negative adolescent developmental outcomes triggered by peer aggression are more diverse, including cognitive, affective, and behavioral dimensions. In addition to the mediating role of self-control between peer victimization and risk-taking behavior and the moderating role of positive parenting, the influence of other variables cannot be excluded. Future studies could further explore the role of variables at different levels, which could help to propose more systematic interventions for prevention and treatment.

## 6. Conclusions

Drawing on social–cognitive theory and the person–environment interaction model, this study presents a more comprehensive illustration of the impact of peer victimization on adolescent risk-taking behavior. The mediation analysis revealed a mediating mechanism of self-control by which peer victimization increased risk-taking behavior. The moderated mediation analysis indicated that positive parenting played a moderating role in attenuating the negative impact of self-control on risk-taking behavior.

## Figures and Tables

**Table 1:** The means, standard deviations, and correlation among the variables.

	1	2	3	4	5	6	7	8	9	10
Covariates										
1. Adolescent age at T1	-									
2. Adolescent gender	−0.03	-								
3. Parent age at T1	0.38 **	−0.06	-							
4. Parent gender	−0.04	0.06	−0.20 **	-						
5. Father’s education	−0.07	0.02	0.02	0.04	-					
6. Mother’s education	−0.08	0.02	−0.07	0.13 **	0.44 ***	-				
Key variables										
7. T1 Peer Victimization	0.01	−0.11 *	0.02	−0.01	0.09 *	−0.04	-			
8. T2 Self-Control	−0.25 ***	0.01	−0.09	0.03	−0.05	0.01	−0.21 ***	-		
9. T2 Positive Parenting	−0.04	−0.05	−0.01	0.01	0.06	0.12 **	−0.06	0.21 ***	-	
10. T2 Risk-Taking Behavior	0.08	−0.15 **	−0.04	−0.05	0.01	−0.01	0.06	−0.26 ***	−0.13 **	-
*M*	14.44	1.50	41.61	1.50	2.11	1.94	0.67	3.22	3.24	0.17
*SD*	1.55	0.50	4.34	0.50	0.53	0.57	0.54	0.61	0.98	0.22
Min	11.59	1.00	35.00	1.00	1.00	1.00	0.00	1.31	1.00	0.00
Max	18.85	2.00	56.00	2.00	4.00	4.00	4.00	4.92	5.00	1.64

Note: Sample size ranged from 448 to 488 due to missing data. * *p* < 0.05, ** *p* < 0.01, *** *p* < 0.001. Adolescent gender: 1 = male, 2 = female; Parent gender: 1 = male, 2 = female; education: 1 = primary school and below, 2 = middle school degree, 3 = undergraduate degree, 4 = postgraduate degree; T1 = Time 1, T2 = Time 2.

**Table 2:** Summary of the direct and indirect effects.

Direct and Indirect Effects	Bias-Corrected Bootstrapped Estimates for the Effects			
	Unstandardized	*SE*	95% CI	Standardized
Direct Pathway				
T1 Peer victimization → T2 Risk-taking behavior	−0.00	0.02	(−0.039, 0.034)	−0.01
Indirect Pathway				
T1 Peer victimization → T2 Self-control → T2 Risk-taking behavior	**0.02**	**0.02**	**(0.025, 0.087)**	**0.05**

Note: T1 = Time 1, T2 = Time 2. The significant results are in bold.

**Table 3:** Summary of the moderated mediation model.

	T2 Self-Control (*R*^2^ = 0.11)			T2 Risk-Taking Behavior (*R*^2^ = 0.2)		
	*B*	*SE*	95% CI	*B*	*SE*	95% CI
Covariates						
Adolescent age at T1	**−0.10**	**0.02**	**(−0.136, −0.067)**	0.00	0.00	(−0.014, 0.013)
Adolescent gender	−0.01	0.06	(−0.123, 0.098)	**−0.08**	**0.02**	**(−0.120, −0.038)**
Father’s level of education	−0.05	0.05	(−0.172, 0.063)	0.00	0.02	(−0.045, 0.050)
Mother’s level of education	−0.00	0.05	(−0.093, 0.093)	0.01	0.02	(−0.035, 0.048)
Study variables						
T1 Peer victimization	**−0.22**	**0.06**	**(−0.336, −0.114)**	−0.01	0.02	(−0.046, 0.033)
T2 Self-control				**−0.09**	**0.02**	**(−0.131, −0.051)**
T2 Positive parenting				**−0.02**	**0.01**	**(−0.044, −0.003)**
T2 Self-control × T2 Positive parenting				**0.04**	**0.02**	**(0.000, 0.075)**

Note: Adolescent gender: 1 = male, 2 = female; education: 1 = primary school and below, 2 = middle school degree, 3 = undergraduate degree, 4 = postgraduate degree; T1 = Time 1, T2 = Time 2. The significant results are in bold.

**Table 4:** Conditional indirect effects of T1 peer victimization on T2 risk-taking behavior via T2 self-control by levels of T2 positive parenting.

Levels of T2 Positive Parenting	Indirect Effect	*SE*	95% CI
Low	**0.03**	**0.01**	**(0.012, 0.053)**
Med	**0.04**	**0.01**	**(0.009, 0.038)**
High	**0.01**	**0.01**	**(0.003, 0.028)**
Diff = High − Low	**−0.02**	**0.01**	**(−0.038, −0.001)**

Note: T1 = Time 1, T2 = Time 2. The significant results are in bold.

## Data Availability

The data presented in this study are available on request from the corresponding authors.
